# Liver Fluke-Derived Molecules Accelerate Skin Repair Processes in a Mouse Model of Type 2 Diabetes Mellitus

**DOI:** 10.3390/ijms252212002

**Published:** 2024-11-08

**Authors:** Anna Kovner, Yaroslav Kapushchak, Oxana Zaparina, Dmitry Ponomarev, Maria Pakharukova

**Affiliations:** 1 Institute of Cytology and Genetics, Siberian Branch of Russian Academy of Sciences (ICG SB RAS), 10 Akad. Lavrentiev Ave., Novosibirsk 630090, Russia; yarikkaps@yandex.ru (Y.K.); zp.oksana.93@gmail.com (O.Z.); p.dmitr@outlook.com (D.P.); pmaria@yandex.ru (M.P.); 2 Department of Natural Sciences, Novosibirsk State University, 2 Pirogova Str., Novosibirsk 630090, Russia

**Keywords:** *Opisthorchis felineus*, excretory–secretory product, egg, skin wound healing, type 2 diabetes mellitus

## Abstract

Chronic nonhealing wounds, such as diabetic ulcers, are among the most serious complications of diabetes mellitus. Consequently, the search for new therapeutic strategies remains highly relevant. Based on our previous data on acute wounds, bioactive molecules derived from the liver fluke *Opisthorchis felineus* hold promise as a novel approach to wound healing. The aim of this study was to investigate the wound-healing properties of excretory–secretory products (ESP) and inactivated eggs of *O. felineus* in a model of type 2 diabetes mellitus. Two-month-old mice of the BKS.Cg + Leprdb/+Leprdb/OlaHsd (db/db) strain were inflicted with superficial wounds of 5 mm in diameter. Mouse groups included several controls (methylcellulose as the vehicle and human recombinant PDGF as the positive control) and specific-treatment groups (ESP and inactivated *O. felineus* eggs). Histopathological, immunohistochemical, and RT-PCR studies using markers for M1/M2 polarization, angiogenesis, and extracellular matrix remodeling were carried out. Additionally, an image analysis of Masson’s trichrome-stained skin sections was performed. The proliferation of HaCaT cells under ESP and egg treatment was also assessed. The present study reveals a significant increase in the percentage of wound healing in ESP- and egg-treated groups, which significantly exceeded the control values after 14 days. Wound treatment with either ESP or worm eggs resulted in (i) a reduction in inflammation with a canonical M1-to-M2 polarization shift, (ii) the modulation of the vascular response, and (iii) dermal extracellular matrix remodeling. All results are comparable to those of the positive control group treated with PDGF. This study also reveals that ESP, but not *O. felineus* eggs, stimulated keratinocyte proliferation in vitro. The results indicate the high wound-healing potential of liver fluke bioactive molecules and open prospects for further research on these new promising therapeutic approaches.

## 1. Introduction

Currently, 1 in 11 adults aged 20 to 79 years has diabetes mellitus, with estimated 537 million cases worldwide [1]. The two major forms of diabetes mellitus are types 1 and 2 [2]. Moreover, approximately 90% of cases of diabetes are type 2 diabetes mellitus (T2DM) [3]. Among all patients with diabetes, more than 25% will develop a nonhealing diabetic foot ulcer during their lifetime [4]. Normally, the dynamics of wound healing consist of four overlapping phases: hemostasis, inflammation, proliferation, and remodeling [5]. The hemostasis stage lasts for the first few hours and, in brief, involves (i) the directed migration, activation, and aggregation of platelets, leading to the formation of a platelet plug; (ii) the activation of the blood coagulation system, resulting in the deposition and stabilization of fibrin, which helps to stop bleeding; and (iii) the subsequent chemoattraction of neutrophils, monocytes, and macrophages [6]. During this stage of wound healing, a release of proinflammatory cytokines and growth factors, such as platelet-derived growth factor (PDGF), epidermal growth factor (EGF), fibroblast growth factor 2 (FGF2), and transforming growth factor-β (TGF-β), is initiated [7].

The inflammation phase lasts for up to 6 days and briefly consist of the following: (i) neutrophils initiate phagocytosis, participate in oxidative burst processes, and generate neutrophil extracellular traps [8]; (ii) monocytes differentiate into macrophages with a predominantly proinflammatory M1 phenotype, which phagocytose cell debris and pathogens while secreting proinflammatory cytokines and matrix metalloproteinases [9]; and (iii) T cells are involved in the immune response and also stimulate the proliferation of keratinocytes by releasing keratinocyte growth factor and insulin growth factor 1 [10,11].

The proliferation phase overlaps with the inflammatory stage and lasts on average more than 14 days: (i) the macrophage phenotype shifts to a reparative M2 phenotype, which promotes both angiogenesis and an increase in granulation tissue by secreting vascular endothelial growth factor (VEGFα), PDGF, and TGF-β [11]; (ii) VEGFα and hypoxia-inducible factor (HIF-1α) stimulate endothelial cell proliferation and migration as well as angiogenesis through three main signaling pathways: PI3K/AKT/mTOR, Jagged1/Notch, and Wnt/β-catenin [12,13]; (iii) keratinocyte activation and migration are also mediated by the PI3K/AKT/mTOR, Jagged1/Notch, and Smad-dependent TGF-β1-mediated signaling pathways [14,15]; and (iv) granulation tissue formation and activation of fibroblasts occur, causing the production of collagen, glycosaminoglycans, proteoglycans, fibronectin, and elastin, which are the major components of the extracellular matrix (ECM) [16].

The remodeling phase can last for 2 weeks to several years and involves a reorganization of granulation tissue: (i) the transition of fibroblasts to myofibroblasts occurs—along with a sequential change from type III to type I secreted collagens (Col1a1)—with the active and balanced participation of matrix metalloproteinases (MMP2 and MMP9) and tissue inhibitor of metalloproteinases 1 (TIMP-1) [17,18]; and (ii) a decrease in the number of endothelial cells, fibroblasts, and macrophages takes place [6]. As a consequence, during the normal course of wound-healing processes, ECM reorganization occurs, giving rise to normal skin architecture.

Patients with diabetes mellitus may develop chronic nonhealing ulcers, which arise as a result of impairments at each stage of wound healing [19]. First, chronic nonhealing wounds are characterized by a nonresolving inflammatory phase, during which large numbers of neutrophils and M1 macrophages, bacterial biofilms, and high levels of proinflammatory cytokines are present in the wound bed [20,21]. Impaired immune cell function has been well documented in patients with diabetes mellitus [22], who exhibit impaired phagocytic activity and leukocyte dysfunction [23]. Another aggravating factor is the persistent state of hypoxia concurrent with angiogenesis failure, which leads to an increase in reactive oxygen species production and dysfunctional wound healing [24]. The suppression of connective tissue growth factors in chronically nonhealing wounds correlates with reduced levels of TGF-β and collagen, and this downregulation slows down wound closure by affecting the balance between fibroblast proliferation and differentiation, the diminished synthesis of ECM components, and a decrease in the vascular cell population in both animal models and humans [25,26,27].

Currently, wound-healing strategies in patients with diabetes mellitus can be categorized into standard and advanced treatments. The standard treatment of diabetic ulcers includes wound care, regular dressing changes, antibiotics in case of infection, and possibly debridement to remove inflamed and/or necrotic tissue [28]. Some of the most common advanced therapies under investigation include hydrogel matrices [29], growth factors, especially PDGF [30], cell therapy [31], and various plant-derived [32] and animal-derived compounds [33,34]. Nevertheless, one of the few FDA-approved agents for diabetic ulcers is currently a drug based on becaplermin (recombinant human PDGF), which promotes granulation tissue formation and wound healing [35]. On the other hand, some data indicate an elevated risk of cancer in patients receiving more than three tubes of becaplermin [36].

A new promising approach to wound healing as well as to diabetes treatment seems to be biologically active secretome molecules and mixed antigens from inactivated trematode eggs [33,37,38,39,40,41]. The trematode *Opisthorchis felineus*, which is widespread in Russia, Kazakhstan, Belarus, and a number of European countries, can be considered a source of biologically active compounds that initiate wound healing [42,43,44,45,46,47]. The disease caused by these parasites is characterized by a prolonged course and ultimately causes serious damage to the hepatobiliary system [48,49,50]. Nonetheless, it has been stated that during chronic *O. felineus* infestation, the integrity of the epithelial layer of cholangiocytes is restored near or directly at the site of parasite attachment [51,52]. Apparently, trematodes have the ability to reduce the acute inflammation caused by helminthic invasion: they initiate the T helper 2 (Th2) immune response and stimulate the restoration of tissues damaged by helminths [53,54,55,56]. They are also able to stimulate skin wound healing and angiogenesis [32,38,39,40,41,53,57,58]. The aim of this study was to test *O. felineus* excretory–secretory products (ESP) and inactivated eggs as skin wound-healing agents in a mouse model of T2DM.

## 2. Results

### 2.1. Glucose Levels and Pancreatic Injury Assessment

**Figure 1:**
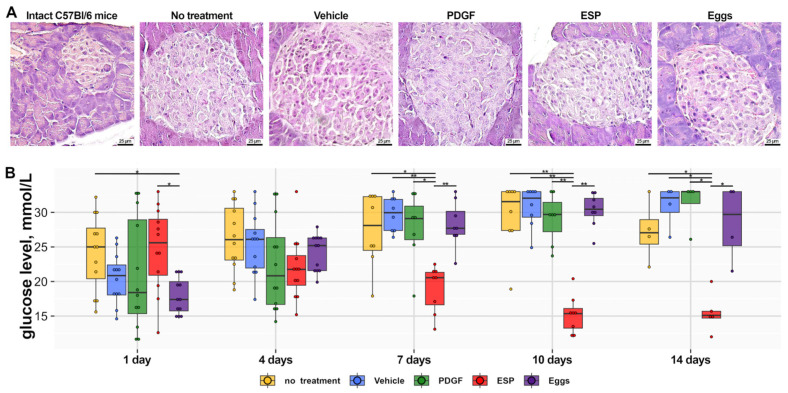
The pancreas and blood glucose in experimental db/db mice. (**A**) Representative pictures of pancreatic islets. Hematoxylin and eosin (H&E) staining, magnification ×400. (**B**) Blood glucose levels (mmol/L). * *p* ≤ 0.05, ** *p* ≤ 0.01.

A histopathological evaluation of pancreatic islets revealed no significant changes in size, cell number, or insulitis among all groups of db/db mice (Figure 1A).

Blood glucose levels (Figure 1B) were above the cutoff value of 14.9 mmol/L in all groups of animals but were significantly lower in the ESP-treated group.

### 2.2. Assessment of the Wound Area

**Figure 2:**
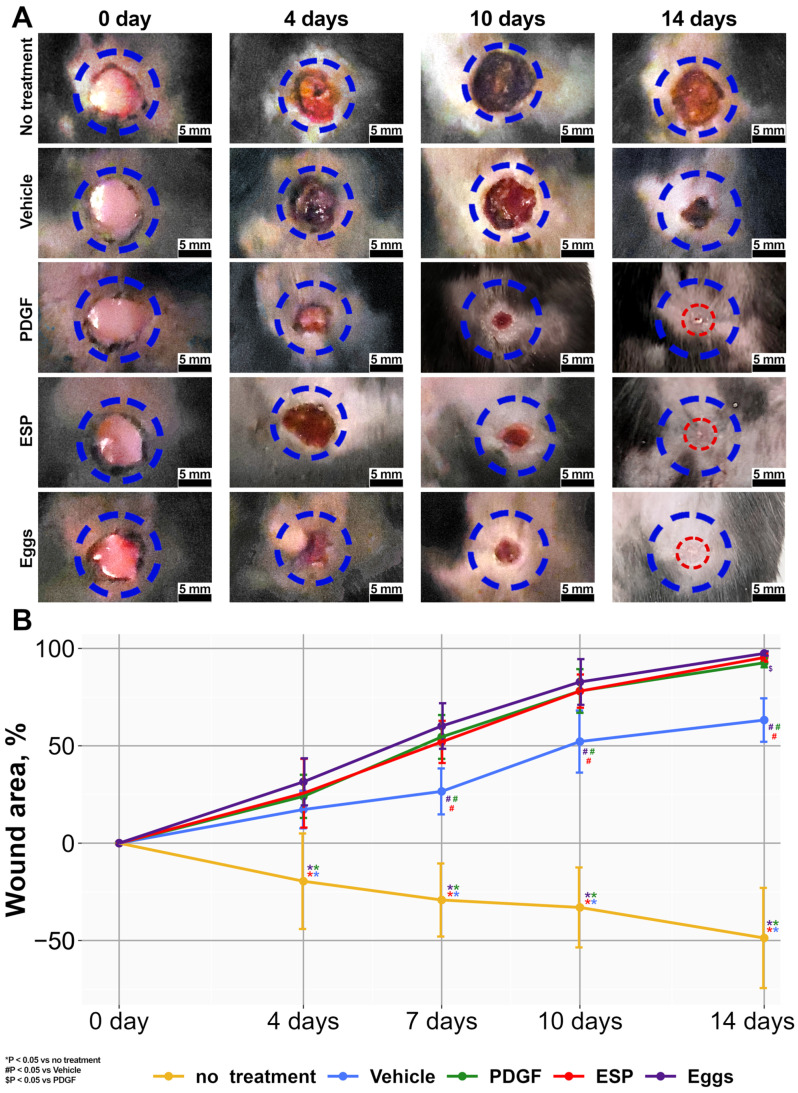
Effects of *O. felineus* ESP and inactivated eggs on diabetic wound healing. (**A**) Representative images of the wound on days 0, 4, 10, and 14 in several groups: without treatment, 1.5% methylcellulose (vehicle), positive control (PDGF), and specific treatment (ESP or Eggs). The wound area is delineated with a dotted line. (**B**) The percentage of wound area (* *p* < 0.05 compared to the “no treatment” group; ^#^ *p* < 0.05 compared to the “vehicle”; ^$^ *p* < 0.05 compared to the “PDGF”).

The application of either ESP (ESP group) or inactivated eggs (egg group) significantly improved wound closure in the mouse model of T2DM (Figure 2A) compared to animals in the vehicle (1.5% methylcellulose) group. It should be noted that there was no difference between the specific-treatment groups and the PDGF-positive control group. By day 14 of the experiment, the extent of wound closure was more than 98% in the specific-treatment groups and positive-control groups. In the group of animals without treatment, the wound area enlarged over time (Figure 2B).

### 2.3. Inflammation Phase Assessment

After confirmation that ESP and worm eggs possess wound-healing potential, the detection of wound-healing processes was carried out. Skin tissue was analyzed for key processes of the three main phases—inflammation, proliferation, and/or remodeling—at 4, 10, and 14 days after treatment.

Semiquantitative analysis of hematoxylin and eosin-stained slides showed that in the vehicle group on the 14th day of the experiment, all tissue samples taken from the wound area remained at the proliferation stage: the presence of a wet crust and epithelial ridges was registered. In both specific-treatment groups and in the PDGF group, wound tissue samples were at the stage of remodeling: a lack of wet crust as well as the initial processes of re-epithelialization (closing of epithelial ridges) were detected (Table 1).

**Figure 3:**
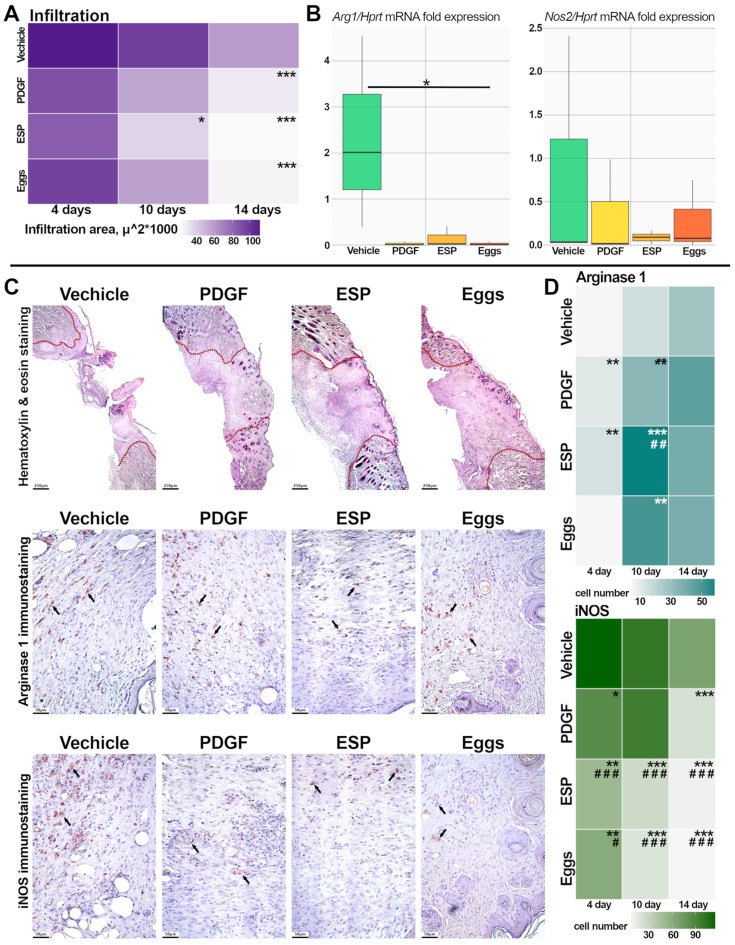
Evaluation of the inflammation phase of wound healing. (**A**) Histopathological analysis of the area of an inflammatory infiltrate (mm^2^) is presented as a heat map. (**B**) mRNA levels of genes *Arg1* and *Nos2* were normalized to the mRNA level of *Hprt*. Data are presented as mean ± SEM, * *p* ≤ 0.05, *** *p* ≤ 0.001 compared to the vehicle group. (**C**) Representative histological images: wound area, H&E staining, and 14 days after treatment, magnification ×40. The dotted line marks the wound area; IHC staining for arginase-1 and inducible NO synthase, 14 days after treatment, magnification ×200; stain-positive cells are indicated with arrows. (**D**) Histopathological analysis of the number of Arg1- and iNOS-positive cells is presented as a heat map. Data are presented as mean ± SEM, * *p* ≤ 0.05, ** *p* ≤ 0.01, *** *p* ≤ 0.001 compared to the vehicle group; # *p* ≤ 0.05, ## *p* ≤ 0.01, ### *p* ≤ 0.001 compared to the PDGF group.

On day 14 of the experiment, a decrease in the inflammation area (μm^2^) was observed in both specific-treatment groups compared to the vehicle group (Figure 3A). A histological examination of the vehicle group revealed the presence of eschar, epithelial ridges, edema, hemorrhage, and an inflammatory infiltrate. Infiltrative changes were observed in the dermis and hypodermis both at the wound area and at the edges in the zone of conditionally healthy tissue in the vehicle group. In contrast, the specific-treatment groups exhibited minimal infiltration, which was primarily localized to the hypodermis. The lowest magnitude of infiltrative changes was observed in the specific-treatment groups (Figure 3A,C).

In addition, to verify the decrease in the inflammation area, the expression of molecular markers of M1/M2 macrophages (inducible NO-synthase (iNOS/*Nos2*) and arginase1 (Arg1)) was analyzed. Immunohistochemical (IHC) analysis of these markers suggested that the number of iNOS^+^ cells (M1 phenotype) was maximal in the specific-treatment groups on the 4th day of the experiment; in the PDGF group, on the 10th day of the experiment; and in the vehicle group, it remained elevated throughout the entire study period. Moreover, a significant decrease in M1 macrophages in the wound area was observed in both specific-treatment groups compared to the PDGF group. A peak in the number of Arg1^+^ cells (M2 phenotype) was observed on the 10th day of the experiment in the specific-treatment groups, in contrast to the PDGF and vehicle groups (Figure 3C,D). This finding may indicate the successful resolution of the inflammation stage, including a canonical M1-to-M2 polarization switch, in the specific-treatment groups.

The mRNA levels of all selected genes (*Nos2* and *Arg1*) in the specific-treatment groups were comparable to those in the positive-control group PDGF. Moreover, the mRNA level of *Arg1* was significantly higher in the vehicle group (Figure 3B).

### 2.4. Proliferation Phase Evaluation

**Figure 4:**
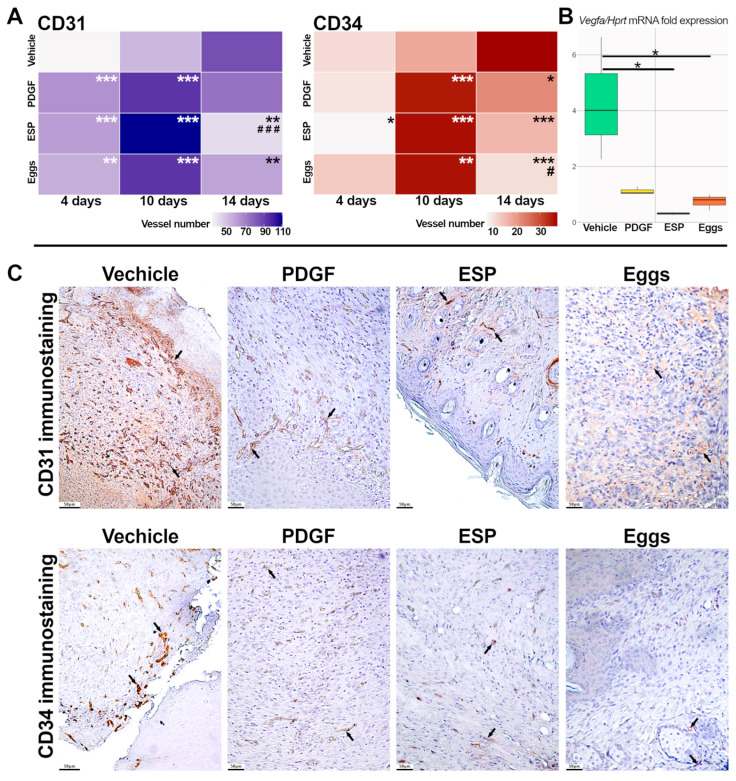
Evaluation of the proliferation stage of wound healing in db/db mice. (**A**) Histopathological analysis of the number of CD31-positive (total) and CD34-positive (young and newly formed) vessels is presented as a heat map. (**B**) The mRNA level of the *Vegfa* gene was normalized to the mRNA level of *Hprt*. Data are presented as mean ± SEM, * *p* ≤ 0.05, ** *p* ≤ 0.01, *** *p* ≤ 0.001 compared to the vehicle group; # *p* ≤ 0.05, ### *p* ≤ 0.001 compared to the PDGF group. (**C**) IHC staining for CD31 and CD34, 14 days after treatment, magnification ×200; stain-positive vessels are marked by arrows.

To assess the ability of ESP and eggs to modify angiogenesis, the number of CD31- and CD34-positive vessels was determined. The maximal number of total vessels (CD31-positive) was observed on day 10 in all groups except the vehicle group. Moreover, a significant decrease in the total number of vessels on day 14 was observed only in the specific-treatment groups: in the egg group compared to the vehicle group, and in the ESP group compared to both control groups (Figure 4A,C).

In the specific-treatment groups and in the PDGF group, the number of CD34-positive newly formed vessels increased by day 10 in contrast to the vehicle group. By day 14, a significant decrease in the number of new and young vessels was also detected in the specific-treatment groups and the PDGF group, indicating an accelerated wound-healing timeline. However, in the egg group, there was also a significant decrease in CD34+ vessels compared to the PDGF group (Figure 4A,C).

On day 14 of the experiment, in both specific-treatment groups, the expression of the *Vegfa* gene was lower than that in the vehicle group (Figure 4B), which may indicate the successful completion of the proliferation stage.

### 2.5. Remodeling Phase Assessment

**Figure 5:**
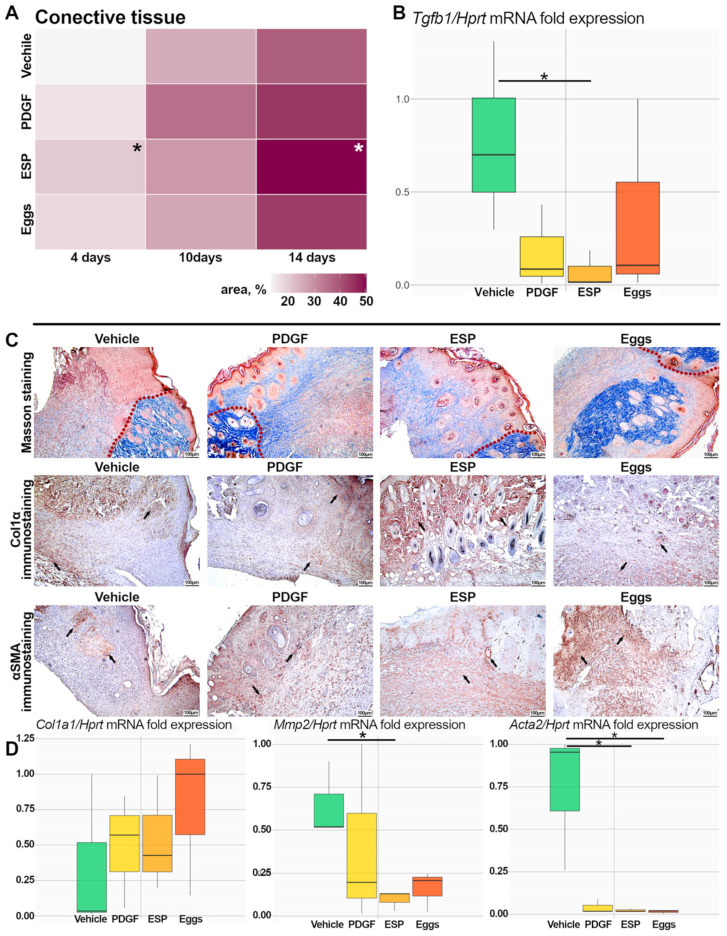
Evaluation of the remodeling stage of wound healing in db/db mice. (**A**) Histopathological analysis of the connective-tissue proportion (%) in the wound area is presented as a heat map. (**B**) The mRNA level of *Tgfb1* was normalized to that of *Hprt*. Data are presented as mean ± SEM, * *p* ≤ 0.05 compared to the vehicle group. (**C**) Representative pictures of connective tissue in the wound area on the 14th day after treatment (Masson staining, collagen fibers are blue), IHC staining for type I collagen and α-smooth muscle actin; the stain-positive area is indicated by arrows); magnification ×100. (**D**) mRNA levels of genes *Col1a1*, *Mmp2*, and *Acta2* were normalized to the mRNA level of the *Hprt* gene. Data are presented as mean ± SEM, * *p* ≤ 0.05 compared to the vehicle group.

The evaluation of the area (%) of connective tissue (Masson’ staining, the collagen fibers are blue) showed that this parameter increased from day 4 to day 14 of the experiment in all groups. Nonetheless, in the wound area of animals from the ESP group, the percentage of connective tissue was higher than in the vehicle group, in contrast to the egg and PDGF groups (Figure 5A,C). This may mean a successful remodeling phase. In the specific-treatment and positive-control groups, tissue samples showed a physiologically normal arrangement of the epidermis and dermis, in contrast to the vehicle group (Figure 3C and Figure 5C).

By day 14 of the experiment, only in the ESP group was the mRNA level of the *Tgfb1* gene significantly different from that in the vehicle group (Figure 5B). This phenomenon was accompanied by the under-expression of the α-smooth muscle actin (*Acta2*) gene in the specific-treatment groups compared to the control group (Figure 5D). The presence of myofibroblasts usually leads to a decrease in wound size, but their excessive number can induce scarring. The visible upregulation of αSMA was noted in both specific-treatment groups (Figure 5C). Additionally, no significant differences in the mRNA level of the *Col1a1* gene (type I collagen) were found between the mouse groups (Figure 5D). It should also be pointed out that there was a visible increase in the deposition of Col1a1^+^ collagen in the ECM in the wound area on day 14 of the experiment in all groups except the vehicle group (Figure 5C).

Only in the ESP group was there significant downregulation of the *Mmp2* (matrix metalloproteinase) gene, which is responsible for ECM remodeling (Figure 5D). Thus, during T2DM, *O. felineus* ESP had a more pronounced effect at the remodeling stage of wound healing.

### 2.6. ESP Increase the HaCaT Cell Number in a Time-Dependent Manner

**Figure 6:**
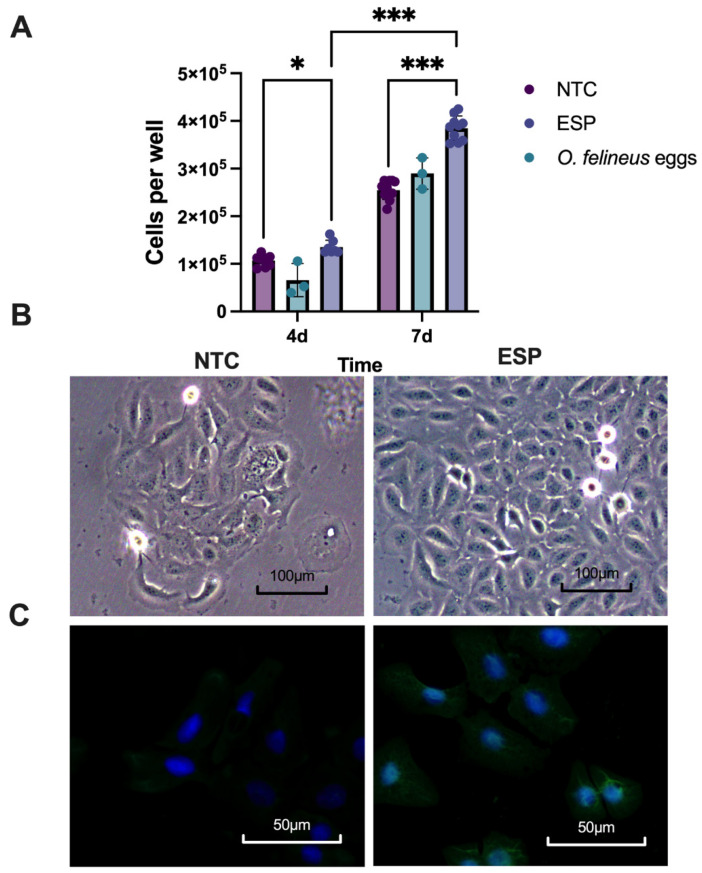
Proliferation of HaCaT cells after treatment with ESP. (**A**) The number of HaCaT cells after incubation with *O. felineus* ESP in the medium with 1% of FBS. Seven replicates were implemented. Data are represented as mean ± SD. (**B**) HaCaT cells after 7 days of cultivation with *O. felineus* ESP, magnification ×100. (**C**) Immunocytochemical analysis of HaCaT cells. Cells stained for an *O. felineus* common antigen (green) and DAPI-labeled nuclei (blue), magnification ×400. NTC: no-treatment control cells; ESP: cells treated with ESP of *O. felineus*. * *p* < 0.033 compared to the no-treatment control group; *** *p* < 0.001 compared to the no-treatment control group.

To assess the mitogenic effect of *O. felineus* ESP, HaCaT cells were cultured with 10 µg/mL ESP for 4 and 7 days. It turned out that after 7 days of cultivation, the number of cells in the ESP-treated group increased by 40% (*p* < 0.001). No significant differences in cell numbers between the groups were observed on day 4. ESP probably exerted a mitogenic effect on HaCaT cells in a time-dependent manner (Figure 6A,B). There was no significant effect of cocultivation with eggs (proliferation increased by 13% on the 7th day of the experiment) (Figure 6A).

In addition, we detected positive staining for the total ESP antigen in the HaCaT cells subjected to ESP treatment (Figure 6C).

## 3. Discussion

This study is the first to demonstrate the wound-healing potential of bioactive molecules from *Opisthorchis felineus* in a T2DM mouse model. The effects of ESP and inactivated *O. felineus* eggs were comparable to those of the positive control, human PDGF (becaplermin), which is FDA-approved for the treatment of diabetic ulcers [59]. Specifically, we observed (i) a significant reduction in wound area; (ii) decreased inflammation; (iii) a canonical change in the number of vessels; and (iv) ECM remodeling. The choice of these treatment groups was based on previous acute-wound model experiments [33] and the extensive literature reporting the high wound-healing potential of soluble egg antigens (SEAs) from *Schistosoma* spp. [40,41,60,61]. Notably, the effects on wound-healing processes were comparable between the ESP and inactivated egg groups.

Numerous studies highlight the potential of bioactive molecules from trematodes as multi-effect agents against diabetes mellitus and its complications [40,41,60,61]. In a T2DM model, soluble egg antigen (SEA) from *Schistosoma japonicum* significantly reduced glucose and insulin levels and enhanced wound healing, likely by stimulating the Th2 immune response and increasing the number of regulatory T cells [40]. In a streptozotocin (STZ)-induced T1DM model, microinjections of SEA from *S. japonicum* reduced blood glucose levels and improved pancreatic health, including the attenuation of insulitis severity, mainly by balancing Th1/Th2 responses [41]. Additionally, in T1DM and T2DM models involving *S. mansoni* infection and obesity, a significant decrease in blood glucose levels was observed compared to uninfected groups [60]. A decrease in blood glucose levels associated with an increase in the number of pancreatic Langerhans islets was observed using an STZ-induced T1DM with *S. mansoni* infection [62]. In the present study, a significant decrease in blood glucose levels was observed solely in the ESP group, although glucose levels remained above the threshold value. No reliable changes in pancreatic islet size were detected. This differential host response may be attributed to the distinct biologies of the trematode species. Notably, studies on *O. viverrini* granulin (Ov-GRN-1), a component of its ESP, have also indicated high wound-healing potential in an acute-wound model [38,39]. Previously, we demonstrated the wound-healing potential of both lysate and ESP from *O. felineus* in a model of acute wounds in C57BL/6 mice. Since proteomic analysis did not detect granulin in the *O. felineus* lysate and ESP, granulin is likely not the sole protein promoting wound-healing processes [33,63].

To characterize the wound-healing processes, this study evaluated the three main phases: inflammation, proliferation, and remodeling. In this study, we observed a decrease in the area of inflammatory infiltrates in the wound area and a change in macrophage phenotype from M1 (iNOS^+^ staining cells) to M2 (Arginase-1^+^ staining cells). This indicates the successful resolution of the inflammation stage, which is critical for wound-healing processes in diabetes mellitus [64]. Our data align with the known ability of trematodes to modulate an M2 immune response [65,66]. Notably, in the pancreas of mice infected with *Schistosoma mansoni* and simultaneously suffering from STZ-induced T1DM, the expression of the *Nos2* gene decreases while the expression of *Arg1* increases [62]. Consistent with these findings, both specific-treatment groups in our study exhibited decreased inflammation, a reduced number of iNOS+ cells, and an elevated number of Arginase-1+ cells.

The key process during the proliferation phase is the activation of angiogenesis [67]. The formation of new blood vessels at a site of injury is mainly initiated by Vegfα [68]. Nevertheless, in addition to chronic inflammation, a critical factor in the pathogenesis of chronic wounds in diabetes mellitus is the persistent state of hypoxia resulting from insufficient angiogenesis [69]. It has previously been reported that the ESP and extracellular vesicles of *O. felineus* can stimulate the formation of pseudo-capillaries in vitro and promote angiogenesis in vivo. This has been demonstrated in an acute-wound model in mice, as well as in the liver of infected Syrian hamsters and humans [33,53]. An angiogenic effect has been demonstrated in *O. viverrini* (Ov-GRN-1) and *Schistosoma* spp. in vitro and in vivo [58,70,71]. It has been found that one of the key mechanisms activating angiogenesis during the proliferation stage is the Jagged1/Notch signaling pathway [72]. *O. felineus* and *S. japonicum* have previously been shown to activate the Notch signaling pathway in the liver of both animal models and humans [50,73]. In the current study, a canonical increase followed by a decrease in the total number of vessels (CD31^+^) and the number of newly formed vessels (CD34^+^) was observed in both specific-treatment groups over the course of the experiment. This occurred concurrently with a decrease in the expression of the *Vegfa* gene, corroborating previous reports on the ability of *O. felineus* to modulate angiogenesis.

The proliferation phase is also characterized by the closure of epithelial folds, involving the activation of keratinocytes with the formation of an epithelial barrier [74]. During normal wound-healing processes, keratinocytes located at the wound margins are activated and migrate toward each other to close the wound [68]. The subcutaneous injection of the larval form of *S. mansoni* has been shown to enhance the differentiation and proliferation of epidermal keratinocytes and of hair follicle keratinocytes [75]. This study demonstrates, for the first time, an increase in keratinocyte proliferation during cultivation with *O. felineus* ESP. This finding is consistent with in vivo data, where the ESP group exhibited reduced crust formation and complete re-epithelialization by day 14. Nevertheless, no significant increase in keratinocyte proliferation was detected during coculturing with *O. felineus* eggs. In the egg treatment group, epithelial closure was observed on day 14 of the in vivo experiment. The discrepancy between the in vivo and in vitro effects in the egg group may be due to a cascade of indirect mechanisms, including the switching of macrophages to the M2 phenotype and activation of the TGF-β pathway. This is because keratinocyte activation not only determines the restoration of the epithelial barrier but also influences the formation of granulation tissue by participating in the balance of pro- and anti-inflammatory responses [76].

According to the “hygienic” hypothesis, parasitic worms have evolved the ability to manipulate host immunity to protect themselves from elimination and minimize host pathology [77]. For instance, the ability to switch the immune response from Th1 to Th2 is also associated with the activation of genes responsible for fibrogenesis and the remodeling stage in wound healing [54,78,79]. Additionally, a recombinant anti-inflammatory protein, AIP-1, derived from the ESP of *Ancylostoma caninum*, has been shown to modulate anti-inflammatory cytokines such as IL-10 and TGF-β in a mouse colitis model. This represents another potential mechanism by which the sequential phases of wound healing may be stimulated [80].

In this study, the under-expression of the *Acta2* gene was detected, suggesting the successful completion of the remodeling stage. A previous study has shown that wound treatment with a TGF-β mimic obtained from *Heligmosomoides polygyrus* ESP results in *Acta2* gene downregulation, with a general beneficial impact on ECM reorganization processes [81]. At the remodeling phase, however, some differences were observed between the specific-treatment groups: in the ESP group, mRNA levels of *Tgfb1* and *Mmp2* were significantly lower than in the egg group. The observed differences may be attributed to the pleiotropic effects of TGF-β, including its post-translational influence on the extracellular matrix [82]. Increased levels of MMPs delay the wound-healing process and cause uncontrolled destruction of the existing or newly deposited extracellular matrix [83]. Thus, the observed decrease in *Mmp2* gene expression in both acute and chronic wounds may indicate canonical wound healing in response to treatment with bioactive molecules derived from *O. felineus* [33]. A large number of bioactive proteins and/or microRNAs (as components of ESP) that specifically affect the remodeling phase may explain this difference. Notably, among the egg-secreted proteins of *S. mansoni*, the presence of an interleukin-4-inducing protein precursor (IPSE/ALPHA-1) has been identified in a major fraction [84], and IL-4-accelerated wound closure in mice has been reported [85].

**Figure 7:**
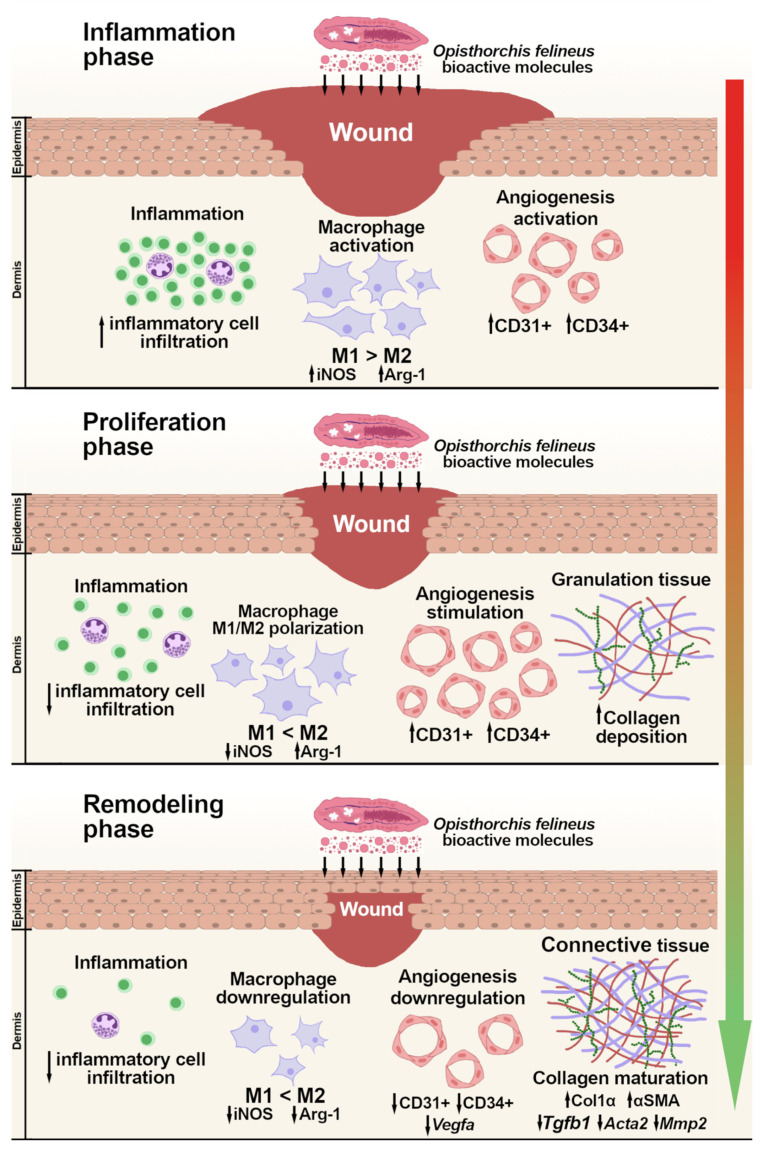
Schematic illustrating the potential cascade of wound-healing reactions of bioactive molecules from the liver fluke *Opisthorchis felineus* in a mouse model of type 2 diabetes mellitus (prepared using GIMP 2.10, https://www.gimp.org/). ↓: Indicates a decrease in the number of positive cells and/or a decrease in the expression of the specified gene. ↑: Indicates an increase in the number of positive cells and/or an increase in the expression of the specified gene.

During the proliferation stage, fibroblast differentiation into myofibroblasts is activated, primarily driven by the TGF-β1 signaling pathway. Myofibroblasts, through the production of a large amount of α-SMA, acquire the ability to contract and reduce the wound area. In this study, we observed an apparent increase in α-SMA-positive cells and fibers within the wound area in all specific-treatment groups. Consequently, during the subsequent remodeling process, the TGF-β signaling pathway also stimulates the production of type I and type III collagens in fibroblasts. Over time, type III collagen is gradually replaced by type I collagen through regulation by matrix metalloproteinases [86]. On the other hand, on the 14th day of the experiment, we did not observe significant differences in the expression of the *Col1a1* gene, despite a simultaneous noticeable increase in type I collagen in the wound area in the specific-treatment groups. It has been demonstrated earlier that trematodes *O. felineus* and *Clonorchis sinensis* activate pathways responsible for ECM remodeling in the liver, including the TGF-β pathway, ECM–receptor interaction, and activities of cell adhesion molecules [54,87]. The successful activation in the remodeling phase in specific-treatment groups may also be related to the activation of these groups of genes. In summary, the results we obtained are presented in Figure 7.

## 4. Materials and Methods

### 4.1. Ethical Statement

All procedures were conducted in compliance with EU Directive 2010/63/EU for animal experiments. Study design protocols and standard operating procedures (concerning hamsters, mice, and fish) were approved by the Ethics Committee on Animal Experiments at the ICG SB RAS [permit number 25 of 12 December 2014 (approval for hamsters and fishes) and permit number 155 of 11 September 2023 (approval for mice)]. All methods are reported in accordance with ARRIVE guidelines (https://arriveguidelines.org). Hamsters and mice were examined daily for signs of illness, injury, or abnormal behavior by the animal facility’s trained personnel. Food and water availability and the macroenvironment (temperature, humidity, noise, light intensity, and cleanliness) were evaluated daily. The animals were fed a standard autoclaved rodent diet, specifically ssniff^®^ R/M-H V1534 (Soest, Germany), which was approved by the specialist overseeing the SPF Animal Facility of the Institute of Cytology and Genetics. No unexpected animal deaths were registered during this study.

### 4.2. Parasites, Animals, and Experimental Design

#### 4.2.1. *O. felineus* Metacercariae

Metacercariae of *O. felineus* were collected from naturally infected *Leuciscus idus* fish from the Ob River (Novosibirsk, Western Siberia, Russia) and isolated from muscle tissues as previously described and described in Supplementary Materials S2 Table S3 [49].

#### 4.2.2. *O. felineus* Adult Worms

Syrian hamsters (n = 7), obtained from the Conventional Animal Facility at ICG SB RAS (Novosibirsk, Russia), were infected with 75 metacercariae. After 3 months, the hamsters were euthanized via carbon dioxide inhalation for 4 min. Worms were isolated from the gallbladder and hepatic bile ducts. Viable worms were manually selected under a binocular light microscope and subsequently washed several times with sterile saline (0.9% NaCl).

#### 4.2.3. ESP

The excretory–secretory products (ESP) were obtained from adult *Opisthorchis felineus* individuals (n = 150). The complete protocol has been described in detail previously and is also outlined in Supplementary Materials S2 [33].

#### 4.2.4. Inactivated Eggs

Eggs were collected from a culture medium [RPMI (Thermo Scientific, Waltham, MA, USA), 1% of glucose, 100 µg/mL streptomycin (Sigma-Aldrich, St. Louis, MO, USA), 100 IU/mL penicillin (Sigma-Aldrich, USA)] of adult *O. felineus*. Finally, the medium with eggs was centrifuged, and the precipitate was frozen at −80 °C. All the above operations were performed under aseptic conditions. The *O. felineus* eggs were lyophilized into powder after being frozen for a week and then sterilized by UV as described elsewhere [41].

#### 4.2.5. Wound Healing in the Murine Model of T2DM

Two-month-old mice of the BKS.Cg + Leprdb/+Leprdb/OlaHsd (db/db) strain (average weight 35–40 g) were obtained from the Specific Pathogen-Free Animal Facility at the ICG SB RAS (Novosibirsk, Russia). All mice were housed in standard individually ventilated cages with unrestricted access to food and water. Mice were anesthetized with isoflurane [2% isoflurane with oxygen (O_2_) at 1 L/min], their back hair was shaved, and superficial wounds with a diameter of 5 mm were inflicted using a stencil. Next, the animals were randomly distributed into the following five groups:

1. Wounded without treatment (n = 12);
2. Vehicle (1.5% methylcellulose (Sigma-Aldrich, lot #SLCF9694, USA) (n = 15) (V);
3. Positive control (0.1% human recombinant PDGF (ProSpec, lot # CYT-501, Rehovot, Israel) (n = 15) (PDGF);
4. Specific treatment: ESP without endotoxin 10 µg (n = 15) (ESP group) or inactivated eggs 40 µg (n = 15) (egg group).

**Figure 8:**
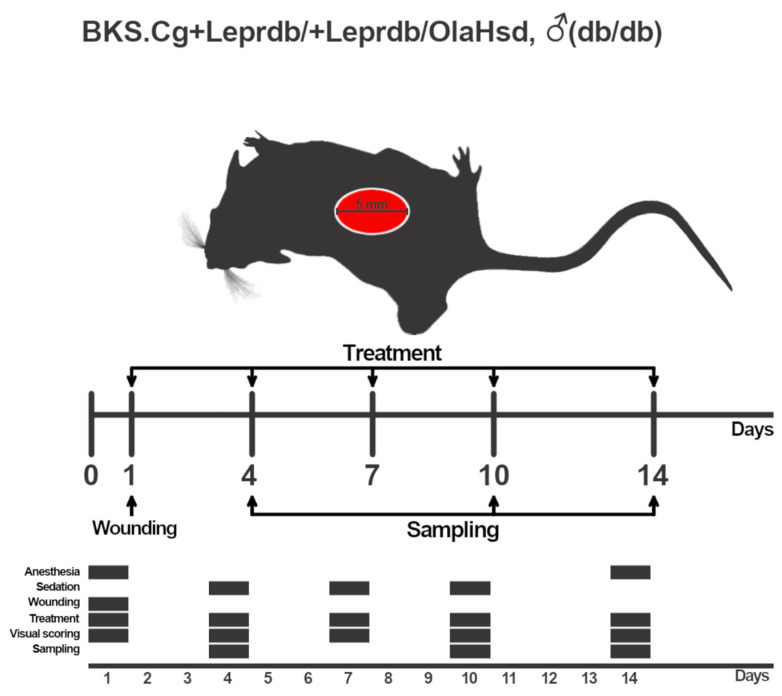
Schematic presentation of the experimental design. Each BKS.Cg + Leprdb/+Leprdb/OlaHsd (db/db) mouse (n = 48) was inflicted a superficial wound (5 mm in diameter). The entire duration of the experiment was 14 days, during which the animals were anesthetized twice and received sedation thrice. All groups received treatment every 3 days with simultaneous measurement of the wound area. Mice were withdrawn from the experiment on days 4, 10, and 14 of treatment with simultaneous collection of damaged-skin samples for histological examination and for gene expression analysis.

The experiment lasted 14 days. The animals were treated every 3 days, with the simultaneous measurement of the wound area on special lined backing (BioVitrum, Saint-Petersburg, Russia). The experimental scheme is presented in Figure 8.

In each group, the treatment of wounds was carried out according to the following scheme:

1. The application of a substance (for all groups, the test substance was placed in a 1.5% methylcellulose solution in PBS (Sigma-Aldrich, lot #SCLF9694, USA));
2. The application of a Luxplast liquid plaster spray (Farmac-zabban, Calderara di Reno BO, Italy).

This protocol is standard and has been validated [33,57]. The Vehicle group was chosen as a reference group (nonspecific control) [33].

The wound tissue was divided into 2 parts: (1) a part of tissue was fixed in 10% aqueous neutral formalin (BioVitrum, Saint-Petersburg, Russia), and (2) the other part of tissue was immediately placed in an RNA-later solution and stored at −20 °C for subsequent RNA isolation (Synthol, Moscow, Russia). The sampling was carried out on days 4, 10, and 14 after the wounding (Figure 8).

A 4-stage blinded study protocol was employed: the first researcher divided the groups of animals based on randomization and was aware of the treatment each animals received; the second researcher administered anesthesia and monitored the animals; the third researcher performed all surgical interventions and selected material for research; the fourth researcher assessed wound areas, described morphological changes, and analyzed gene expression.

### 4.3. Wound Scoring

Every 3 days (Figure 8), the animals were photographed on a special lined backing (BioVitrum, Russia). Subsequently, the wound area in the photographs was measured using ImageJ software (version number 1.50i, https://imagej.net/). The extent (%) of wound healing was determined for each mouse individually.

The extent of wound healing was calculated using the following formula:

$$\%\mathrm{wound}\mathrm{healing}=100\%-\frac{\mathrm{wound}\mathrm{area}\mathrm{on}\mathrm{the}\mathrm{day}\mathrm{of}\mathrm{measurement},{\mathrm{mm}}^{2},\times 100\%}{\mathrm{wound}\mathrm{area}\mathrm{on}\mathrm{the}\mathrm{first}\mathrm{day}\mathrm{of}\mathrm{measurement},{\mathrm{mm}}^{2}}$$

### 4.4. Histopathological Assessment

A histological assessment of skin samples was performed as previously described, and it is detailed in Supplementary Materials S2 [33].

The resulting paraffin sections were stained via a standard protocol with H&E and the Mallory dye (detecting connective tissue fibers). To determine the stage of wound healing, an IHC analysis was performed (Immunohistochemical SpringBioScience Kit HRP-125, Pleasanton, CA, USA) using specific primary antibodies to analyze the following:

1. Inflammation: Arg1 (Abcam, cat. # ab233548, 1:100, Waltham, MA, USA) and iNOS (Santa Cruz Biotechnology, cat. #F1113, 1:100, Dallas, Texas, USA).
2. Neoangiogenesis: CD34 (Abcam, cat. # ab81289, 1:300) and CD31 (Affinity, cat. # AF8016, 1:100, Buckingham, United Kingdom).
3. State of the ECM: collagen I (Abcam, cat. # ab34710, 1:200) and α-smooth muscle actin (α-SMA) (Abcam, cat. # ab7817, 1:300).

The staining was performed according to the manufacturers’ protocols and as detailed in Supplementary Materials S2. The visualization was carried out under an AxioImager A1 microscope (Zeiss, Germany) with a AxioCam MRc camera (Zeiss, Germany). Semi-quantitative analysis on histological sections was performed to assess the presence of wet crust (“+/−”) and epithelial ridges (“+/−”). The proportion (%) of an infiltrate in an area was determined in μm^2^ using ImageJ software (version number 1.50i, https://imagej.net/). By means of a closed test system for 100 points (Morphometry and ImageJ software), the proportion (%) of connective tissue, numbers of Arg1- and iNOS-positive cells, and numbers of CD31- and CD 34-positive blood vessels in the wound area were determined.

### 4.5. Gene Expression Analysis

Total RNA was isolated using ExtractRNA (Evrogen, Moscow, Russia) as previously described and detailed in Supplementary Materials S2 [33].

### 4.6. Proliferative Activity of HaCaT Cells

HaCaT cells are a commercially available transformed human keratinocyte epithelial cell line (the line was provided by the scientific research institute Cell Culture Collection: a multi-access center at the Institute of Biological Research, the Russian Academy of Sciences). The cells were cultured in the DMEM/F12 medium (Sigma-Aldrich, USA) with 10% of FBS (Gibco, Waltham, MA, USA), an antibiotic–antimycotic solution (Gibco, USA), and 2 mM L-glutamine (Gibco, USA) at 37 °C and 5% CO_2_. A 0.25% trypsin solution (Gibco, USA) was utilized to detach cells from culture plastic. The cells were subcultured at 1:5 upon reaching 80–90% confluence.

HaCaT cells were seeded in a 6-well plate at a concentration of 20,000/well. The isolated ESP were added to the cells at a concentration of 10 μg/mL, eggs at a concentration of 10 µg/mL, and BSA as a no-treatment control was added at 10 μg/mL. Cultivation with the ESP or eggs was carried out in the medium with the FBS concentration of 1%.

The cells were treated with the 0.25% trypsin solution and removed from the culture plate. Then, a 0.4% Trypan blue dye solution (Bio-Rad, USA) was added to the cell suspension in a 1:1 ratio. The stained solution along with the cells was transferred to a Goryaev chamber, where live cells were counted under a light microscope. Cells were counted above large squares. The principle of the method is selective staining of damaged cells with trypan blue. At least seven technical replicates were set up. The experiment was repeated two times.

### 4.7. Immunocytochemistry

Immunocytochemical analysis was performed to detect the presence of the *O. felineus* common antigen in cells after cultivation with ESP. For this assay, HaCaT cells were cultivated with ESP under standard conditions at 7 days. The following primary antibodies were employed: a mouse anti-common *O. felineus* antigen–antibody (1:500) and a secondary anti-mouse antibody (1:500, Abcam, ab97046), according to the standard protocol (detailed in Supplementary Materials S2). Images were captured using an AxioCam MRc camera (Carl Zeiss, Oberkochen, Germany) attached to a fluorescence microscope (ZEISS Imager M2).

### 4.8. Statistical Analysis

This analysis was performed using STATISTICA 7.0 (Statsoft, Tulsa, OK, USA), Prism software packages (version 10.3.0., https://www.graphpad.com). For the in vivo experiment, the data were expressed as a percentage of the maximal possible score and presented as a heat map by means of the heatmap.2 (v.3.1.3) R package (https://www.rdocumentation.org/packages/gplots/versions/3.1.3/topics/heatmap.2, accessed on 11 October 2024).

The normality of the data distribution was determined with the Shapiro–Wilk test. Significant differences between the control and experimental groups of mice were evaluated with the pairwise Wilcoxon test for histological data and Dunn’s test for mRNA expression data. Each graph was created using R package ggplot2 (version 3.5.1). A *p* value less than 0.05 was assumed to indicate statistical significance. The results of the pairwise tests are presented in Supplementary Materials S1 Tables S1 and S2. For the in vitro experiment, the Shapiro–Wilk test was used to check the normality of the data distribution. A mixed-effect model (REML) with Sidak’s post hoc test for multiple comparisons was applied.

## 5. Conclusions

While this study demonstrates the beneficial effects of active biomolecules from *Opisthorchis felineus* in type 2 diabetes mellitus, it is crucial to acknowledge that infection with this parasite can lead to various pathologies. Thus, patient acceptance of therapies based on parasitic worms may be psychologically challenging. Nevertheless, inactivated eggs and excretory–secretory products of *O. felineus* show promising potential to enhance wound healing in a T2DM mouse model. Our findings indicate that bioactive molecules in both ESP and inactivated eggs mediate canonical wound-healing processes, including macrophage phenotype switching (M1-to-M2), angiogenesis, and extracellular matrix reorganization. These results are comparable or superior to those observed in the PDGF group, the active component of Regranex ointment approved for clinical use in humans with diabetic ulcers. Further proteomic analysis of inactivated *O. felineus* eggs, the isolation of proteins common to major fractions (ESP, lysate, and eggs), and the identification of other bioactive molecules (including microRNAs), followed by in vitro and in vivo testing, offer promising avenues for the discovery of novel wound-healing agents for chronic non-healing wounds.

## Figures and Tables

**Table 1:** Semiquantitative pathomorphological analysis of cutaneous wound.

Parameters	Vehicle			PDGF			ESP			Eggs		
Days	4	10	14	4	10	14	4	10	14	4	10	14
Wet crust	+	+	+	+	+	-	+	+	-	+	+	-
Epithelial ridge	+	+	+	+	+	-	+	+	-	+	+	-

## Data Availability

The raw data supporting the conclusions of this article can be found in the Supplementary Materials.

## Supplementary Materials

The following supporting information can be downloaded at https://www.mdpi.com/article/10.3390/ijms252212002/s1. Reference [88] is cited in the Supplementary Material.

## References

[1] Hossain M.J., Al-Mamun M., Islam M.R. (2024). Diabetes mellitus, the fastest growing global public health concern: Early detection should be focused. Health Sci. Rep..

[2] Singh R., Gholipourmalekabadi M., Shafikhani S.H. (2024). Animal models for type 1 and type 2 diabetes: Advantages and limitations. Front. Endocrinol..

[3] Ong K.L., Stafford L.K., McLaughlin S.A., Boyko E.J., Vollset S.E., Smith A.E., Dalton B.E., Duprey J., Cruz J.A., Hagins H. (2023). Global, regional, and national burden of diabetes from 1990 to 2021, with projections of prevalence to 2050: A systematic analysis for the Global Burden of Disease Study 2021. Lancet.

[4] Ramirez-Acuña J.M., Cardenas-Cadena S.A., Marquez-Salas P.A., Garza-Veloz I., Perez-Favila A., Cid-Baez M.A., Flores-Morales V., Martinez-Fierro M.L. (2019). Diabetic Foot Ulcers: Current Advances in Antimicrobial Therapies and Emerging Treatments. Antibiotics.

[5] MacLeod A.S., Mansbridge J.N. (2016). The Innate Immune System in Acute and Chronic Wounds. Adv. Wound Care.

[6] Rodrigues M., Kosaric N., Bonham C.A., Gurtner G.C. (2019). Wound Healing: A Cellular Perspective. Physiol. Rev..

[7] Guo S., Dipietro L.A. (2010). Factors affecting wound healing. J. Dent. Res..

[8] Wilgus T.A., Roy S., McDaniel J.C. (2013). Neutrophils and Wound Repair: Positive Actions and Negative Reactions. Adv. Wound Care.

[9] Chen C., Yang J., Shang R., Tang Y., Cai X., Chen Y., Liu Z., Hu W., Zhang W., Zhang X. (2024). Orchestration of Macrophage Polarization Dynamics by Fibroblast-Secreted Exosomes during Skin Wound Healing. J. Invest. Dermatol..

[10] Tanno H., Kawakami K., Ritsu M., Kanno E., Suzuki A., Kamimatsuno R., Takagi N., Miyasaka T., Ishii K., Imai Y. (2015). Contribution of Invariant Natural Killer T Cells to Skin Wound Healing. Am. J. Pathol..

[11] Tanno H., Kawakami K., Kanno E., Suzuki A., Takagi N., Yamamoto H., Ishii K., Imai Y., Maruyama R., Tachi M. (2017). Invariant NKT cells promote skin wound healing by preventing a prolonged neutrophilic inflammatory response. Wound Repair. Regen..

[12] Zhang D., Lv F.L., Wang G.H. (2018). Effects of HIF-1α on diabetic retinopathy angiogenesis and VEGF expression. Eur. Rev. Med. Pharmacol. Sci..

[13] Zheng X., Narayanan S., Sunkari V.G., Eliasson S., Botusan I.R., Grünler J., Catrina A.I., Radtke F., Xu C., Zhao A. (2019). Triggering of a Dll4-Notch1 loop impairs wound healing in diabetes. Proc. Natl. Acad. Sci. USA.

[14] Chigurupati S., Arumugam T.V., Son T.G., Lathia J.D., Jameel S., Mughal M.R., Tang S.-C., Jo D.-G., Camandola S., Giunta M. (2007). Involvement of notch signaling in wound healing. PLoS ONE.

[15] Ding Y., Ma S.-Q., Li M., Chen L., Feng S.-M. (2024). Promoter Hypermethylation Downregulates MiR-125b-5p and MiR-199b-5p Targeting of ΔNp63, Resulting in PI3K/AKT/mTOR Pathway Activation and Keratinocyte Differentiation. Comb. Chem. High. Throughput Screen..

[16] Zhang T., Wang X.-F., Wang Z.-C., Lou D., Fang Q.-Q., Hu Y.-Y., Zhao W.-Y., Zhang L.-Y., Wu L.-H., Tan W.-Q. (2020). Current potential therapeutic strategies targeting the TGF-β/Smad signaling pathway to attenuate keloid and hypertrophic scar formation. Biomed. Pharmacother..

[17] Boraldi F., Lofaro F.D., Bonacorsi S., Mazzilli A., Garcia-Fernandez M., Quaglino D. (2024). The Role of Fibroblasts in Skin Homeostasis and Repair. Biomedicines.

[18] Naduk-Kik J., Hrabec E. (2008). The role of matrix metalloproteinases in the pathogenesis of diabetes mellitus and progression of diabetes retinopathy. Postepy. Hig. Med. Dosw..

[19] Baltzis D., Eleftheriadou I., Veves A. (2014). Pathogenesis and treatment of impaired wound healing in diabetes mellitus: New insights. Adv. Ther..

[20] Lin C.W., Hung C.M., Chen W.J., Chen J.C., Huang W.Y., Lu C.S., Kuo M.L., Chen S.G. (2022). New Horizons of Macrophage Immunomodulation in the Healing of Diabetic Foot Ulcers. Pharmaceutics.

[21] Tkaczyk C., Jones-Nelson O., Shi Y.Y., Tabor D.E., Cheng L., Zhang T., Sellman B.R. (2022). Neutralizing Staphylococcus aureus Virulence with AZD6389, a Three mAb Combination, Accelerates Closure of a Diabetic Polymicrobial Wound. mSphere.

[22] Richard C., Cristall L., Fleming E., Lewis E.D., Ricupero M., Jacobs R.L., Field C.J. (2017). Impact of Egg Consumption on Cardiovascular Risk Factors in Individuals with Type 2 Diabetes and at Risk for Developing Diabetes: A Systematic Review of Randomized Nutritional Intervention Studies. Can. J. Diabetes.

[23] Sandoval-Schaefer T., Phan Q., Dash B.C., Prassinos A.J., Duan K., Gazes M.I., Vyce S.D., Driskell R., Hsia H.C., Horsley V. (2023). Transcriptional heterogeneity in human diabetic foot wounds. bioRxiv.

[24] Catrina S.B., Zheng X. (2016). Disturbed hypoxic responses as a pathogenic mechanism of diabetic foot ulcers. Diabetes Metab. Res. Rev..

[25] Park L.K., Maione A.G., Smith A., Gerami-Naini B., Iyer L.K., Mooney D.J., Veves A., Garlick J.A. (2014). Genome-wide DNA methylation analysis identifies a metabolic memory profile in patient-derived diabetic foot ulcer fibroblasts. Epigenetics.

[26] Sutcliffe J.E.S., Thrasivoulou C., Serena T.E., Madden L., Richards T., Phillips A.R.J., Becker D.L. (2017). Changes in the extracellular matrix surrounding human chronic wounds revealed by 2-photon imaging. Int. Wound J..

[27] Voza F.A., Huerta C.T., Le N., Shao H., Ribieras A., Ortiz Y., Atkinson C., Machuca T., Liu Z.J., Velazquez O.C. (2024). Fibroblasts in Diabetic Foot Ulcers. Int. J. Mol. Sci..

[28] Alam W., Hasson J., Reed M. (2021). Clinical approach to chronic wound management in older adults. J. Am. Geriatr. Soc..

[29] Tsourdi E., Barthel A., Rietzsch H., Reichel A., Bornstein S.R. (2013). Current aspects in the pathophysiology and treatment of chronic wounds in diabetes mellitus. Biomed. Res. Int..

[30] Mahmoudvand G., Karimi Rouzbahani A., Razavi Z.S., Mahjoor M., Afkhami H. (2023). Mesenchymal stem cell therapy for non-healing diabetic foot ulcer infection: New insight. Front. Bioeng. Biotechnol..

[31] Zhang H.M., Yang M.L., Xi J.Z., Yang G.Y., Wu Q.N. (2023). Mesenchymal stem cells-based drug delivery systems for diabetic foot ulcer: A review. World J. Diabetes.

[32] Chaudhary R. (2023). The Role of Medicinal Plants in the Diabetic Wound Healing Process. Curr. Diabetes Rev..

[33] Kovner A.V., Tarasenko A.A., Zaparina O., Tikhonova O.V., Pakharukova M.Y., Mordvinov V.A. (2022). Wound healing approach based on excretory-secretory product and lysate of liver flukes. Sci. Rep..

[34] El-Tantawy N.L. (2015). Helminthes and insects: Maladies or therapies. Parasitol. Res..

[35] Gilligan A.M., Waycaster C.R., Milne C.T. (2018). Cost Effectiveness of Becaplermin Gel on Wound Closure for the Treatment of Pressure Injuries. Wounds.

[36] Papanas N., Maltezos E. (2010). Benefit-risk assessment of becaplermin in the treatment of diabetic foot ulcers. Drug Saf..

[37] Botelho M.C., Alves H., Richter J. (2016). Wound healing and cancer progression in *Opisthorchis viverrini* associated cholangiocarcinoma. Parasitol. Res..

[38] Bansal P.S., Smout M.J., Wilson D., Cobos Caceres C., Dastpeyman M., Sotillo J., Seifert J., Brindley P.J., Loukas A., Daly N.L. (2017). Development of a Potent Wound Healing Agent Based on the Liver Fluke Granulin Structural Fold. J. Med. Chem..

[39] Dastpeyman M., Bansal P.S., Wilson D., Sotillo J., Brindley P.J., Loukas A., Smout M.J., Daly N.L. (2018). Structural Variants of a Liver Fluke Derived Granulin Peptide Potently Stimulate Wound Healing. J. Med. Chem..

[40] Tang C.L., Yu X.H., Li Y., Zhang R.H., Xie J., Liu Z.M. (2019). Schistosoma japonicum Soluble Egg Antigen Protects Against Type 2 Diabetes in Lepr db/db Mice by Enhancing Regulatory T Cells and Th2 Cytokines. Front. Immunol..

[41] Huang H., Hu D., Chen Z., Xu J., Xu R., Gong Y., Fang Z., Wang T., Chen W. (2022). Immunotherapy for type 1 diabetes mellitus by adjuvant-free Schistosoma japonicum-egg tip-loaded asymmetric microneedle patch (STAMP). J. Nanobiotechnology.

[42] Pakharukova M.Y., Mordvinov V.A. (2022). Similarities and differences among the Opisthorchiidae liver flukes: Insights from *Opisthorchis felineus*. Parasitology.

[43] Fedorova O.S., Fedotova M.M., Zvonareva O.I., Mazeina S.V., Kovshirina Y.V., Sokolova T.S., Golovach E.A., Kovshirina A.E., Konovalova U.V., Kolomeets I.L. (2020). *Opisthorchis felineus* infection, risks, and morbidity in rural Western Siberia, Russian Federation. PLoS Negl. Trop. Dis..

[44] Kiyan V.S., Bulashev A.K., Katokhin A.V. (2018). *Opisthorchis felineus* and *Metorchis bilis* Metacercariae in Cyprinid Fish *Leuciscus idus* in Nura-Sarysu River, Kazakhstan. Korean J. Parasitol..

[45] Scaramozzino P., Condoleo R., Martini E., Bossu T., Aquilani S., Spallucci V., Aquilini E., Marozzi S. (2018). Behaviour and eating habits as determinants for human opisthorchiasis in the Bolsena Lake area, Italy. Folia Parasitol (Praha).

[46] Pozio E., Armignacco O., Ferri F., Gomez Morales M.A. (2013). *Opisthorchis felineus*, an emerging infection in Italy and its implication for the European Union. Acta Trop..

[47] Skripova L.V. (2013). Contamination of water objects with helminth eggs and protozoan cysts in the Minsk Region. Med. Parazitol..

[48] Maksimova G.A., Pakharukova M.Y., Kashina E.V., Zhukova N.A., Kovner A.V., Lvova M.N., Katokhin A.V., Tolstikova T.G., Sripa B., Mordvinov V.A. (2017). Effect of *Opisthorchis felineus* infection and dimethylnitrosamine administration on the induction of cholangiocarcinoma in Syrian hamsters. Parasitol. Int..

[49] Kovner A.V., Pakharukova M.Y., Maksimova G.A., Mordvinov V.A. (2019). Characteristics of liver fibrosis associated with chronic *Opisthorchis felineus* infection in Syrian hamsters and humans. Exp. Mol. Pathol..

[50] Kovner A., Zaparina O., Kapushchak Y., Minkova G., Mordvinov V., Pakharukova M. (2022). Jagged-1/Notch Pathway and Key Transient Markers Involved in Biliary Fibrosis during *Opisthorchis felineus* Infection. Trop. Med. Infect. Dis..

[51] Gouveia M.J., Pakharukova M.Y., Laha T., Sripa B., Maksimova G.A., Rinaldi G., Brindley P.J., Mordvinov V.A., Amaro T., Santos L.L. (2017). Infection with *Opisthorchis felineus* induces intraepithelial neoplasia of the biliary tract in a rodent model. Carcinogenesis.

[52] Zaparina O., Kovner A., Petrova V., Kolosova N., Mordvinov V., Pakharukova M. (2024). Plastoquinone-Derivative SkQ1 Improved the Biliary Intraepithelial Neoplasia during Liver Fluke Infection. Curr. Issues Mol. Biol..

[53] Ponomarev D.V., Lishai E.A., Kovner A.V., Kharkova M.V., Zaparina O., Kapuschak Y.K., Mordvinov V.A., Pakharukova M.Y. (2023). Extracellular vesicles of the liver fluke *Opisthorchis felineus* stimulate the angiogenesis of human umbilical vein endothelial cells. Curr. Res. Parasitol. Vector Borne Dis..

[54] Pakharukova M.Y., Zaparina O., Baginskaya N.V., Mordvinov V.A. (2022). Global changes in gene expression related to *Opisthorchis felineus* liver fluke infection reveal temporal heterogeneity of a mammalian host response. Food Waterborne Parasitol..

[55] Kaewraemruaen C., Sermswan R.W., Wongratanacheewin S. (2016). Induction of regulatory T cells by *Opisthorchis viverrini*. Parasite Immunol..

[56] Zhao L., Shi M., Zhou L., Sun H., Zhang X., He L., Tang Z., Wang C., Wu Y., Chen T. (2018). *Clonorchis sinensis* adult-derived proteins elicit Th2 immune responses by regulating dendritic cells via mannose receptor. PLoS Negl. Trop. Dis..

[57] Smout M.J., Laha T., Mulvenna J., Sripa B., Suttiprapa S., Jones A., Brindley P.J., Loukas A. (2009). A granulin-like growth factor secreted by the carcinogenic liver fluke, *Opisthorchis viverrini*, promotes proliferation of host cells. PLoS Pathog..

[58] Haugen B., Karinshak S.E., Mann V.H., Popratiloff A., Loukas A., Brindley P.J., Smout M.J. (2018). Granulin Secreted by the Food-Borne Liver Fluke *Opisthorchis viverrini* Promotes Angiogenesis in Human Endothelial Cells. Front. Med..

[59] Nguyen T.T., Ding D., Wolter W.R., Pérez R.L., Champion M.M., Mahasenan K.V., Hesek D., Lee M., Schroeder V.A., Jones J.I. (2018). Validation of Matrix Metalloproteinase-9 (MMP-9) as a Novel Target for Treatment of Diabetic Foot Ulcers in Humans and Discovery of a Potent and Selective Small-Molecule MMP-9 Inhibitor That Accelerates Healing. J. Med. Chem..

[60] Amer A.S., Othman A.A., Dawood L.M., El-Nouby K.A., Gobert G.N., Abou Rayia D.M. (2023). The interaction of *Schistosoma mansoni* infection with diabetes mellitus and obesity in mice. Sci. Rep..

[61] Tang C.L., Lian Z., Ding F.R., Liang J., Li X.Y. (2024). Schistosoma-related molecules as a new strategy to combat type 1 diabetes through immune regulation. Parasitol. Int..

[62] Osada Y., Fujiyama T., Kamimura N., Kaji T., Nakae S., Sudo K., Ishiwata K., Kanazawa T. (2017). Dual genetic absence of STAT6 and IL-10 does not abrogate anti-hyperglycemic effects of *Schistosoma mansoni* in streptozotocin-treated diabetic mice. Exp. Parasitol..

[63] Pakharukova M.Y., Savina E., Ponomarev D.V., Gubanova N.V., Zaparina O., Zakirova E.G., Cheng G., Tikhonova O.V., Mordvinov V.A. (2023). Proteomic characterization of *Opisthorchis felineus* exosome-like vesicles and their uptake by human cholangiocytes. J. Proteom..

[64] Li M., Hou Q., Zhong L., Zhao Y., Fu X. (2021). Macrophage Related Chronic Inflammation in Non-Healing Wounds. Front. Immunol..

[65] He Q., Pan X., Yin Y., Xu A., Yi X., Wu Y., Li X. (2023). *Clonorchis sinensis* granulin promotes malignant transformation of human intrahepatic biliary epithelial cells through interaction with M2 macrophages via regulation of STAT3 phosphorylation and the MEK/ERK pathway. Parasit. Vectors.

[66] Bility M.T., Sripa B. (2014). Chronic *Opisthorchis viverrini* infection and associated hepatobiliary disease is associated with iron loaded M2-like macrophages. Korean J. Parasitol..

[67] Ma H., Siu W.S., Leung P.C. (2023). The Potential of MSC-Based Cell-Free Therapy in Wound Healing-A Thorough Literature Review. Int. J. Mol. Sci..

[68] Smith J., Rai V. (2024). Novel Factors Regulating Proliferation, Migration, and Differentiation of Fibroblasts, Keratinocytes, and Vascular Smooth Muscle Cells during Wound Healing. Biomedicines.

[69] Burgess J.L., Wyant W.A., Abdo Abujamra B., Kirsner R.S., Jozic I. (2021). Diabetic Wound-Healing Science. Medicina.

[70] Hembasat T., Chaiyadet S., Ittiprasert W., Smout M.J., Young N.D., Loukas A., Brindley P.J., Laha T. (2023). Peptide derived from progranulin of the carcinogenic liver fluke, *Opisthorchis viverrini* stimulates cell hyperproliferation and proinflammatory cytokine production. Res. Sq..

[71] Mbanefo E.C., Agbo C.T., Zhao Y., Lamanna O.K., Thai K.H., Karinshak S.E., Khan M.A., Fu C.-L., Odegaard J.I., Saltikova I.V. (2020). IPSE, an abundant egg-secreted protein of the carcinogenic helminth *Schistosoma haematobium*, promotes proliferation of bladder cancer cells and angiogenesis. Infect. Agent. Cancer.

[72] Pitulescu M.E., Schmidt I., Giaimo B.D., Antoine T., Berkenfeld F., Ferrante F., Park H., Ehling M., Biljes D., Rocha S.F. (2017). Dll4 and Notch signalling couples sprouting angiogenesis and artery formation. Nat. Cell Biol..

[73] Zheng S., Zhang P., Chen Y., Zheng S., Zheng L., Weng Z. (2016). Inhibition of Notch Signaling Attenuates Schistosomiasis Hepatic Fibrosis via Blocking Macrophage M2 Polarization. PLoS ONE.

[74] Gonzalez A.C.D.O., Costa T.F., de Araújo Andrade Z., Medrado A.R.A.P. (2016). Wound healing—A literature review. An. Bras. Dermatol..

[75] Bourke C.D., Prendergast C.T., Sanin D.E., Oulton T.E., Hall R.J., Mountford A.P. (2015). Epidermal keratinocytes initiate wound healing and pro-inflammatory immune responses following percutaneous schistosome infection. Int. J. Parasitol..

[76] Fernández-Guarino M., Hernández-Bule M.L., Bacci S. (2023). Cellular and Molecular Processes in Wound Healing. Biomedicines.

[77] Qiu S., Fan X., Yang Y., Dong P., Zhou W., Xu Y., Zhou Y., Guo F., Zheng Y., Yang J.Q. (2017). *Schistosoma japonicum* infection downregulates house dust mite-induced allergic airway inflammation in mice. PLoS ONE.

[78] Pinlaor S., Prakobwong S., Boonmars T., Wongkham C., Pinlaor P., Hiraku Y. (2009). Effect of praziquantel treatment on the expression of matrix metalloproteinases in relation to tissue resorption during fibrosis in hamsters with acute and chronic *Opisthorchis viverrini* infection. Acta Trop..

[79] Arunsan P., Chaidee A., Cochran C.J., Mann V.H., Tanno T., Kumkhaek C., Smout M.J., Karinshak S.E., Rodpai R., Sotillo J. (2020). Liver fluke granulin promotes extracellular vesicle-mediated crosstalk and cellular microenvironment conducive to cholangiocarcinoma. Neoplasia.

[80] Ferreira I.B., Pickering D.A., Troy S., Croese J., Loukas A., Navarro S. (2017). Suppression of inflammation and tissue damage by a hookworm recombinant protein in experimental colitis. Clin. Transl. Immunol..

[81] Lothstein K.E., Chen F., Mishra P., Smyth D.J., Wu W., Lemenze A., Kumamoto Y., Maizels R.M., Gause W.C. (2024). Helminth protein enhances wound healing by inhibiting fibrosis and promoting tissue regeneration. Life Sci. Alliance.

[82] Gumede D.B., Abrahamse H., Houreld N.N. (2024). Targeting Wnt/β-catenin signaling and its interplay with TGF-β and Notch signaling pathways for the treatment of chronic wounds. Cell Commun. Signal.

[83] Aldaghi N., Kamalabadi-Farahani M., Alizadeh M., Salehi M. (2024). Doxycycline-loaded carboxymethyl cellulose/sodium alginate/gelatin hydrogel: An approach for enhancing pressure ulcer healing in a rat model. J. Biomed. Mater. Res. A.

[84] Abdulla M.H., Lim K.C., McKerrow J.H., Caffrey C.R. (2011). Proteomic identification of IPSE/alpha-1 as a major hepatotoxin secreted by *Schistosoma mansoni* eggs. PLoS Negl. Trop. Dis..

[85] Salmon-Her V., Ramont L., Godeau G., Birembaut P., Guenounou M., Bernard P., Maquart F.X. (2000). Implication of interleukin-4 in wound healing. Lab. Invest..

[86] Diller R.B., Tabor A.J. (2022). The Role of the Extracellular Matrix (ECM) in Wound Healing: A Review. Biomimetics.

[87] Yoo W.G., Kang J.M., Lê H.G., Pak J.H., Hong S.J., Sohn W.M., Na B.K. (2020). Bile Ductal Transcriptome Identifies Key Pathways and Hub Genes in *Clonorchis sinensis*-Infected Sprague-Dawley Rats. Korean J. Parasitol..

[88] Mordvinov V.A., Minkova G.A., Kovner A.V., Ponomarev D.V., Lvova M.N., Zaparina O., Romanenko S.A., Shilov A.G., Pakharukova M.Y. (2021). A tumorigenic cell line derived from a hamster cholangiocarcinoma associated with *Opisthorchis felineus* liver fluke infection. Life Sci..

