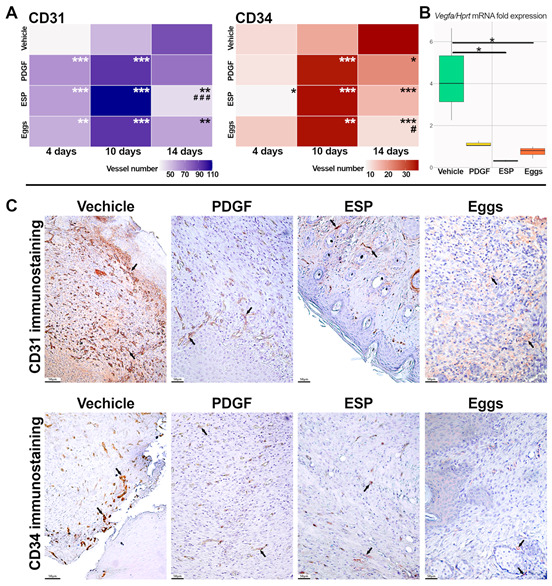# Correction: Kovner et al. Liver Fluke-Derived Molecules Accelerate Skin Repair Processes in a Mouse Model of Type 2 Diabetes Mellitus. *Int. J. Mol. Sci.* 2024, 25, 12002

**DOI:** 10.3390/ijms26136046

**Published:** 2025-06-24

**Authors:** Anna Kovner, Yaroslav Kapushchak, Oxana Zaparina, Dmitry Ponomarev, Maria Pakharukova

**Affiliations:** 1 Institute of Cytology and Genetics, Siberian Branch of Russian Academy of Sciences (ICG SB RAS), 10 Akad. Lavrentiev Ave., Novosibirsk 630090, Russia; 2 Department of Natural Sciences, Novosibirsk State University, 2 Pirogova Str., Novosibirsk 630090, Russia

In the original publication [1], there was a mistake in the Figure 4C. A technical error occurred during the creation of the collage. The correct figure appears below. The authors state that the scientific conclusions are unaffected. This correction was approved by the Academic Editor. The original publication has also been updated.