# Supplementary material for: Liver Fluke-Derived Molecules Accelerate Skin Repair Processes in a Mouse Model of Type 2 Diabetes Mellitus
Source: Int J Mol Sci. 2024 Nov 8;25(22):12002. doi: 10.3390/ijms252212002 (PMC11593665; doi:10.3390/ijms252212002)
Supplement: Supplementary file 1 [file ijms-25-12002-s001.zip › Supplementary Material S2.pdf]

Supplementary Material S2

1. *O. felineus* metacercariae

Metacercariae of *O. felineus* were collected from naturally infected *Leuciscus idus* fish from the Ob River (Novosibirsk, Western Siberia) and isolated from muscle tissues as previously described. The fish were collected from neither conservation areas nor private property and are not otherwise protected officially; hence, fishing permits were not required. *L. idus* is not considered endangered or rare, and the fishing methods complied with Federal Law N166-F3 of 20 December 2004 (18 July 2011 version) "Fishing and conservation of water bio-resources" [1].

2. ESP

The excretory-secretory products (ESP) were obtained from adult *Opisthorchis felineus* individuals (n = 150). Adult worms were kept in a 6-well plate at 37 °C with 5% CO2 for 24 h, in the medium: RPMI (Thermo Scientific, USA), 1% glucose, 100 μg/ml streptomycin (Sigma-Aldrich, USA), 100 IU/ml penicillin (Sigma-Aldrich, USA). After incubation, the medium was collected and the secretory product was isolated.

Isolation steps:

(1) to remove cellular and tissue debris, the medium was centrifuged at 4 °C, 300–500g for 10 min;

(2) supernatant was centrifuged at 4 °C, 2000g for 20 min;

(3) supernatant was centrifuged at 4000g for 10 min at 4 °C;

(4) supernatant was filtered through a filter with a pore size of 0.22 μm (MF-Millipore, USA);

(5) filtrate was loaded into a centrifuge concentrator (MWCO = 5 kDa), centrifuged at 4 °C, 6000g for 2 h;

(6) for solution dialysis, sterile cold PBS (sodium phosphate buffer) was added to the supernatant, then placed in a centrifuge concentrator (MWCO = 5 kDa), centrifuged at 4 °C, 6000g for 30 min;

(7) a cocktail of protease inhibitors (#80-6501-23, GE Healthcare, USA) was added to the concentrate;

(8) concentrate was aliquoted, stored at − 80 °C.

All samples further undergone endotoxin removal using Pierce High Capacity Endotoxin Removal Resin according to the manufacturer's instructions. Protein concentrations were measured spectrophotometrically using a commercial BCA Protein Assay kit (#23225, ThermoScientific, USA) according to the manufacturer's recommendations [2].

3. Histopathological assessment

Histological assessment of skin samples was performed as previously described [2]. Skin samples exposed to 10% neutral formalin were dehydrated in a graded series of ethanol and in xylene (STP-120, ThermoScientific, USA). Dehydrated samples were enclosed in a paraffin medium HISTOMIX (BioVitrum, Russia). For microscopic

examination, sections of 3.5 μm thickness were prepared on a rotary microtome Microm HM 355S (ThermoScientific, USA). The resulting paraffin sections were stained via a standard protocol with hematoxylin and eosin (detection of skin layers, assessment of wound margins, evaluation of re-epithelialization, measurement of inflammatory infiltrate size, and overall pathomorphological assessment) and Mallory staining (detecting connective tissue fibers in the wound area).

4. Gene expression analysis

Total RNA was isolated using ExtractRNA (Evrogen, Russia) as previously described [2]. Concentrations of RNA were determined on a NanoDrop spectrophotometer (ND1000, NanoDrop Technologies, USA). First-strand cDNA synthesis was performed with the RevertAid Kit (Fermentas, European Union). Expression levels of the genes were measured by real-time PCR with the EVA Green Reagent Mix (Synthol, Russia) in a CFX96 real-time PCR system (Bio-Rad, USA). The *Hprt1* gene was chosen as an endogenous internal control. Triplicate real-time PCRs were performed on each sample. The fold change in a target gene's expression (normalized to the controls) was calculated from threshold cycle values (Ct; CFX96 software). Sequences of all primers and probes (Syntol, Russia) are presented below:

Table S3. Primers and probes used for real-time PCR in this study

Name	Sequence	Length (bp)
*Acta2*_probe	VIC-CGAACTGCCTGACGGGCAGG-BHQ2	20
*Acta2*_F	TTCCTTCGTGACTACTGCCGAG	22
*Acta2*_R	AATGCCTGGGTACATGGTGGT	21
Tgfb_F	GTCACTGGAGTTGTACGGCA	20
Tgfb_R	TCTTCTCTGTGGAGCGTTGA	20
Tgfb_probe	VIC-ACAGGGCTTTCGATTCAGCGCT-BHQ2	22
Mmp2_F	GCCCCCATGAAGCCTTGTTT	20
Mmp2_R	ATAGCGGTACAAGTATGCCTCTG	23
Mmp2_probe	VIC-GCACCGATGGCTACCGCTGG-BHQ2	20
Col1a1_F	GAAGATGTAGGAGTCGAGGGACC	23
Col1a1_R	GGCCTTGGAAACCTTGTGGA	20
Col1a1_probe	VIC-GAGTTTCCGTGCCTGGCCCC-BHQ2	20
Vegfa_F	GCACTGGACCCTGGCTTTAG	20
Vegfa_R	TGATCCGCATGATCTGCATGG	21
Vegfa_probe	VIC-CCAGGCTGCACCCACGACAG-BHQ2	20
Arg1_F	ATGACGTGAGAGACCACGGG	20
Arg1_R	TGCTTCCAACTGCCAGACTGT	21
Arg1_probe	VIC-GCCCACCCCAAACACAGTGCA-BHQ2	21
Nos2_F	TGGTGAAGGGACTGAGCTGTT	21
Nos2_R	CAACGTTCTCCGTTCTCTTGC	21
Nos2_probe	VIC-TGAGGCTCCTCACGCTTGGGT-BHQ2	21
*Hprt1*_F	TCCTTAGTCAAGCGGTACAAC	21
*Hprt1*_R	ATCTGGCCTATATCCAACACTTC	23
Hprt_1_probe	VIC-TGGACAGGACTGAAAGACTTGCTCG-BHQ2	25

5. Immunocytochemistry

Immunocytochemical analysis was performed to detect the presence of the *O. felineus* common antigen in cells after cultivation with ESP. For this assay, HaCaT cells were cultivated with ESP under standard conditions at 7 days. The following primary antibodies were employed: a mouse anti-common *O. felineus* antigen antibody (1:500) and a secondary anti-mouse antibody (1:500, Abcam, ab97046), according to the standard protocol.

The protocol "Immunization and serum-collection procedures for producing custom mouse polyclonal antibodies" (ThermoFisher) was used to obtained a mouse anti-common *O. felineus* antigen antibody and briefly was as follows:

(1) subcutaneous injection of the antigen (excretory–secretory product or lysate; 0.1 mg) with complete Freund's adjuvant (150 µl; first immunization: 1st day);

(2) subcutaneous injection of the antigen (50 g of protein) with incomplete Freund's adjuvant (150 µl; immunization: 21st day, 42nd day);

(3) serum collection to determine the antibody titer (50th day);

(4) subcutaneous injection of the antigen (50 g of protein per 300 µl of saline) with incomplete Freund's adjuvant (150 µl; fourth immunization: 62nd day);

(5) postmortem blood sampling and serum collection (80th day).

Briefly, samples were fixed with 4% paraformaldehyde in PBS for 20 min at room temperature, permeabilized with 0.1% Tergitol™ 15-S-9 (NeoFroxx, Germany) in PBS for 10 min, and blocked with 5% BSA in PBS for 30 min. Primary antibodies were applied and incubated overnight at 4 °C. After washing with PBS, the samples were incubated with appropriate secondary antibodies for 1 h at room temperature. After three washes in PBS, the samples were embedded in a mounting medium (Fluoroshield) containing DAPI (Sigma-Aldrich, lot #D9542, USA) for nuclear staining. Images were captured using an AxioCam MRc camera (Carl Zeiss) attached to a fluorescence microscope (ZEISS Imager M2) [3].

References:

1. Kovner, A.V., Pakharukova, M.Y., Maksimova, G.A., Mordvinov, V.A. Characteristics of liver fibrosis associated with chronic *Opisthorchis felineus* infection in Syrian hamsters and humans. *Exp Mol Pathol* **2019**, *110*, 104274. doi: 10.1016/j.yexmp.2019.104274.

2. Kovner, A.V., Tarasenko, A.A., Zaparina, O., Tikhonova, O.V., Pakharukova, M.Y., Mordvinov, V.A. Wound healing approach based on excretory-secretory product and lysate of liver flukes. *Sci Rep* **2022**, *12(1)*, 21639. doi: 10.1038/s41598-022-26275-y.

3. Mordvinov, V.A., Minkova, G.A., Kovner, A.V., Ponomarev, D.V., Lvova, M.N., Zaparina, O., Romanenko, S.A., Shilov, A.G., Pakharukova, M.Y. A tumorigenic cell line derived from a hamster cholangiocarcinoma associated with *Opisthorchis felineus* liver fluke infection. *Life Sci* **2021**, *277*, 119494. doi: 10.1016/j.lfs.2021.119494.
